# Autoscopic phenomena: case report and review of literature

**DOI:** 10.1186/1744-9081-7-2

**Published:** 2011-01-10

**Authors:** Francesca Anzellotti, Valeria Onofrj, Valerio Maruotti, Leopoldo Ricciardi, Raffaella Franciotti, Laura Bonanni, Astrid Thomas, Marco Onofrj

**Affiliations:** 1 Department of Neuroscience and Imaging, Aging Research Centre, Ce.S.I., "G. d'Annunzio" University Foundation G. d'Annunzio University, Chieti, Italy; 2 University Ospedale San Raffaele, Milan, Italy

## Abstract

**Background:**

Autoscopic phenomena are psychic illusory visual experiences consisting of the perception of the image of one's own body or face within space, either from an internal point of view, as in a mirror or from an external point of view. Descriptions based on phenomenological criteria distinguish six types of autoscopic experiences: autoscopic hallucination, he-autoscopy or heautoscopic proper, feeling of a presence, out of body experience, negative and inner forms of autoscopy.

**Methods and results:**

We report a case of a patient with he-autoscopic seizures. EEG recordings during the autoscopic experience showed a right parietal epileptic focus. This finding confirms the involvement of the temporo-parietal junction in the abnormal body perception during autoscopic phenomena. We discuss and review previous literature on the topic, as different localization of cortical areas are reported suggesting that out of body experience is generated in the right hemisphere while he-autoscopy involves left hemisphere structures.

## Background

The term autoscopy comes from the Greek words "*autos*" (self) and "*skopeo*" (looking at). Autoscopic phenomena are psychic illusory visual experiences defined by the perception of the images of one's own body or one's face within space, either from an internal point of view, as in a mirror or from an external point of view. Autoscopic experiences were first described by the Greek philosopher Aristotele, but it was subsequently admirably described in its ambiguous presentation by Ovid in the third book of its *Metamorphoses *where the author narrates the myth of Narcissus, a beautiful boy who falls in love with his image reflected in a water source. In the first place he mistakes his own image with another person, but when he suddenly realizes that he is looking at the image of himself, desperate for the hopelessness of his love he is forced to commit suicide.

The variable presentations of the autoscopic phenomena, which will be extensively addressed below in their scientific meaning and correlates, clearly appear in the comparison between the aforementioned myth of Narcissus and the description made by Plautus in his *Amphitryon*: here the double (represented by the god Mercury) is an entity completely distinct from the character Sosia who shares with his double only the physical aspect, but not the psychological and intellective attitudes. At difference with Narcissus, Sosia is frightened by the double: he believes that the double could have stolen his identity or, worse, that the appearance of the double could have been caused by the death. As it appears from the descriptions the theme of the death is often strictly linked with the theme of the double. Autoscopic phenomena are considered at the basis of many self-portrait paintings [1]: Durer, Rembrandt, Velazquez, Schiele seem to have painted during autoscopic experiences.

Dostoevsky made this phenomenon more popular in the nineteenth century in his novel "The Double", where the doppelganger (i.e. haunting double of the self) of German legends was the background.

The German word "Doppelganger" was brought into the language, and simultaneously, into the literary tradition by the novelist Jean Paul Richter, who in 1796 defined the word in a one sentence footnote: "So heissen Leute, die sich selbst sehen" (So people who see themselves are called).

The first medical report dates back to Wigan's description in 1844. Subsequently, the term autoscopy was used to describe various phenomena of various aetiologies and mechanisms.

Brugger was one of the first authors to study autoscopic phenomena [2]. He proposed a classification scheme based on phenomenological criteria. Along with examples of illustrative cases, he outlined the main features of six types of autoscopic phenomena: the feeling of a presence, the negative heautoscopy, the inner heautoscopy, the autoscopic hallucination, the out of body experience and the he-autoscopy also defined as heautoscopic proper.

The feeling of a presence, first described by Jasper in 1913, is the distinct feeling of the physical presence of another person or being in the near extracorporeal space. No visual impressions are involved but frequently the presence is felt to be "at the fringe of vision". Like he-autoscopy, it is usually accompanied by alterations in the experience of one's own body. It can be reported by healthy persons in conditions of sensory deprivation or social isolation [3]. The feeling of a presence is often confined to one hemispace particularly when associated with a seizure disorder [4].

Negative heautoscopy refers to the failure to perceive one's own body either in a mirror or when looked at directly. In the latter form the confinement of the (negative) hallucination to one's own body and the frequent coexistence of depersonalisation are considered evidences of its close relatedness with the positive forms of autoscopic experiences.

In inner heautoscopy, a type of experience frequently treated by French Authors of the early 20th century [5], the inner organs of one's own body are visually hallucinated in the extra corporal space.

In the French psychiatry literature of the late 19th and early 20th centuries, the typical autoscopic hallucination was also labelled as "mirror hallucination" [6-10] and consists of a visual perception of an exact mirror image of oneself, occasionally only one's face or trunk is perceived. Patients with autoscopic hallucination do not localize themselves at the position of the illusory body. Commonly, autoscopic hallucination lasts only a few minutes or seconds, often followed by flash-like reoccurrences.

In out of body experience, localization of the psychological self to an extrapersonal space is completely dissociated from the perception of one's body, implementing the "dissociation of egocentric" from "body-centred-perspectives" [11,12]. In this form of autoscopic phenomena the subject sees himself and the world from a location different from his physical body (parasomatic visuo-spatial perspective, disembodied location). People explaining their out of body experience never use the words "double" or "doppelgänger" in their descriptions. An out of body experience is defined by the presence of three phenomenological characteristics: disembodiment (location of the self outside one's body), the impression of seeing the world from a distant and elevated visuo-spatial perspective (extracorporeal egocentric perspective) and the impression of seeing one's own body (or autoscopy) from this elevated perspective [11].

The theme of the separation between self and body is frequently found in philosophy. Hume claimed that "when he introspected, he was unable to catch his self without a perception and was unable to observe 'anything but the perception' itself". He concluded that the self (or the observing introspective subject) is simply a collection of different perceptions. The interest of occidental philosophy for the body was also exemplified in Descartes' effort to separate mind and body.

The he-autoscopy is rare but probably more frequent than pure autoscopic hallucinations. The term "Heautoskopie" was proposed by Menninger-Lerchenthal [13] to denote the experience of seeing one's self and to designate the classical doppelgänger experience described in literary accounts [14,15]. The double usually appears colorless ("foggy", "pale", or "as through a veil"), can behave autonomously, may or may not mirror the person's appearance and maintains sidedness. The expression heautoscopic "echopraxia" means imitation of bodily movements by the double, giving rise to the illusion that the doppelgänger contains the real mind [16,17]. Among autoscopic phenomena, "polyopic" [18,19] and "heterosexual" [20] cases have also been published. There is considerable variation in the reported duration of heautoscopic experiences; they may last for seconds or hours and even cases of persistence of the double as a steady companion are not exceptional [21]. Depersonalization may be experienced as a feeling of strangeness towards one's own body or as the impression that one's own mind is contained by the doppelganger, as in the heautoscopic echopraxia. As in out of body experience, patients describe the image of the self as a highly realistic experience. In addition to mere visual impressions, as in autoscopic hallucination, he-autoscopy involves somaesthetic and vestibular sensations. A feeling of detachment or of extreme lightness of one's body is regularly present and often vertigo is reported. Patients describing heautoscopic experiences always reveal significant changes in the awareness of their body, generally do not report clear disembodiment but they are often unable to localize their selves. He-autoscopy is the encounter with one's own "doppelganger" who appears as an alter ego; patients may experience themselves to be localized at the position of the illusory body (bilocation); it is difficult to decide for the patient whether he/she is disembodied or not and whether the self is localized within the physical body or in the autoscopic body. The ego, however, remains localized within the natural boundaries of one's own body. Partial epilepsy, particularly parietal and temporal lobe seizures, is considered the most frequent aetiology [22-24], but autoscopic phenomena may also occur in patients with psychiatric disorders and migraine [11,16]. Conrad [21] described an unusual case of autoscopic phenomenon associated with a tumour of the hypophysis. Other authors reported similar episodes in various organic diseases [10,11,16,17] and some evidenced precipitating factors and neural correlates [12]. Descriptions of out of body experience are often reported in patients undergoing resuscitation procedures [25,26].

To date the central mechanism for autoscopic phenomena are not completely understood. The more convincing hypothesis is a failure to integrate multisensory signals at the temporo-parietal junction, resulting in a breakdown of the spatial unity between self and the body. While the contribution of visual and somatosensory cues of self-location is largely attested by clinical and experimental data [27-29], little is known about the contribution of vestibular cues.

With the present report we aim to discuss and review previous literature on the topic and we present a case of a woman experiencing with he-autoscopy during epileptic seizures. We could record a video-EEG during one of the ictal episodes. During the recording we took notes of the patient impressions. We had the opportunity to describe a rare semiology of he-autoscopy, accompanied by suicidal behaviour and depression and to link it with a specific brain activity pattern.

### Case report

The patient is a right-handed, 40 year old woman employed as a teacher in primary school. She was referred to our neurological centre for sporadic generalized epileptic seizures and reactive depressive syndrome. Early medical history reported depressive-apathetic episodes during adolescence and early adulthood lasting for some months and treated with benzodiazepines. The first generalized seizure occurred at the age of 26 and was accompanied by attention and memory deficits for one week. Three further generalized seizures occurred in 9 years and interictal EEGs showed widespread slow wave activity. She reported several unsuccessful therapeutic attempts with carbamazepine (1000 mg/day) and fenobarbital (150 mg/day). Personality disorder with several cyclic episodes of depression and maniacal excitation were reported by the patient and in the past ten years she had been treated with paroxetine by outpatient psychiatric services. In the last six years she did not experience further seizures and she spontaneously decided to withdraw carbamazepine. In the last year further episodes of depression and anxiety had recurred, and were accompanied by two suicide attempts by poisoning. After the last suicidal attempt, an EEG was recorded in the intensive care unit and the observation of widespread slow wave activity prompted her referral to our clinic. Her cognitive performances were normal (Mini-Mental State Examination score: 30/30). During psychological assessment, beyond a logorrheic and anxious attitude, a history of peculiar events could be evicted even though the patient manifested initial severe resistance to express the reason of her distress. She reported to experience daily symptoms consisting of seeing the image of her entire body as in a mirror or from an external point of view. She saw herself not from an elevated visuo-spatial perspective, as in out of body experience, but in front of her in normal size and colour without a definable facial expression. The patient could not clearly define her localization in space. She reported unclear changes in the awareness of her body describing herself as projected out of her body with a feeling of dissociation of mind and body for a few seconds. When she saw her double from an external view she maintained sidedness, i.e. right and left sides were represented as in the real body, unlike images reflected by a mirror: if she held an object with the right hand her autoscopic image would hold the same object with the right hand. Her heautoscopic experience lasted for less than one minute. These experiences occurred independently of daily activities, either when she was quiet alone or working. When the double appeared, it kept acting the patient's activities. She explained that the experience to see her double was terrifying and that the attempted suicides were prompted by this distressing experience. She reported to have access to the autoscopic body's thoughts, words and actions and that the experience of bilocation was petrifying and shocking. She explained that these experiences had occurred since her early adolescence, had never subsided and were still present when she was receiving carbamazepine and fenobarbital. These episodes were interpreted as he-autoscopic seizures.

**Figure 1:**
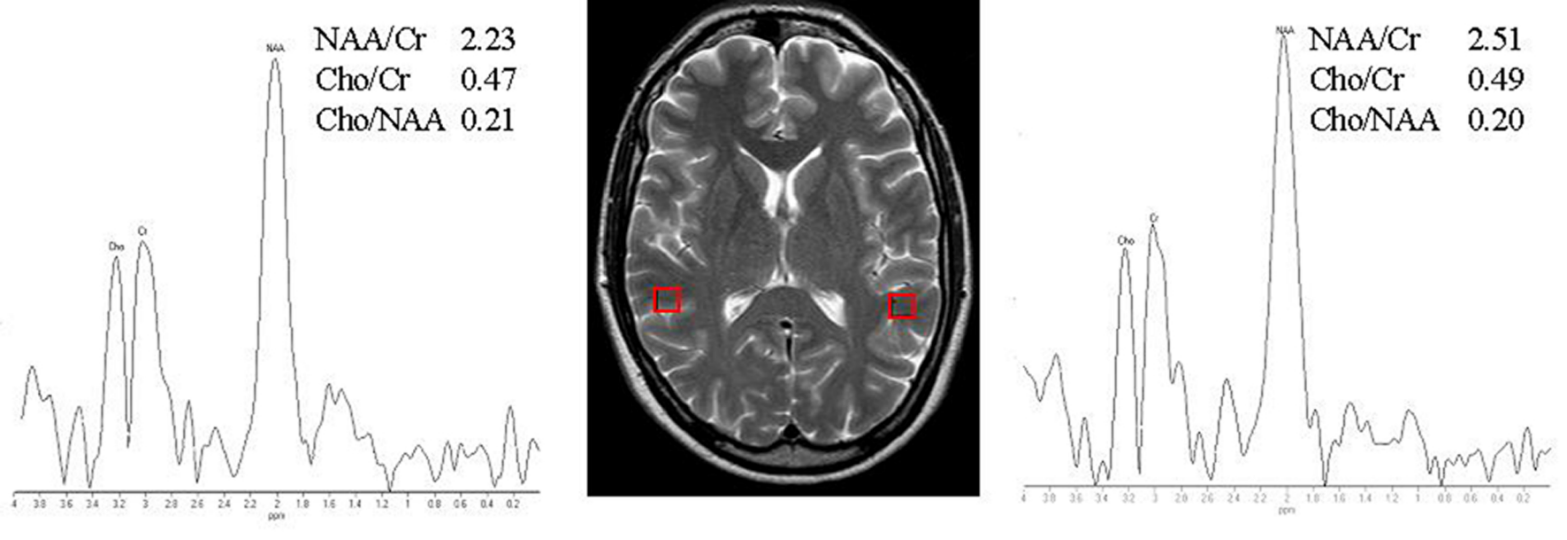
**Proton MR spectra from left and right temporo-parietal junction**. The major metabolite peaks correspond to cholines (Cho) at 3.22 ppm, creatines (Cr) at 3.02 ppm, N-acetylaspartate (NAA) at 2.02 ppm. Axial T2 weighted MR image showing the voxel position at temporo-parietal junctions used for proton MR spectroscopy. Note that the splenium of corpus callosum is normal.

In a previous brain MRI performed in another neurological institution, an abnormal signal from the splenium of corpus callosum was suspected. But an MRI-based tractography performed in our institution showed integrity of white matter tracts. MRI scans were performed using a 3T Philips Achieva scanner. Diffusion tensor images were acquired in the axial plane with diffusion sensitization gradients applied in six non-collinear directions with b-value of 1000s/mm^2^. All image volumes were acquired with six optimized directions using six repetitions to increase the number of measures. In addition NMR spectroscopy evidenced no alteration in the right and left temporo-parietal junction (Figure 1).

**Figure 2:**
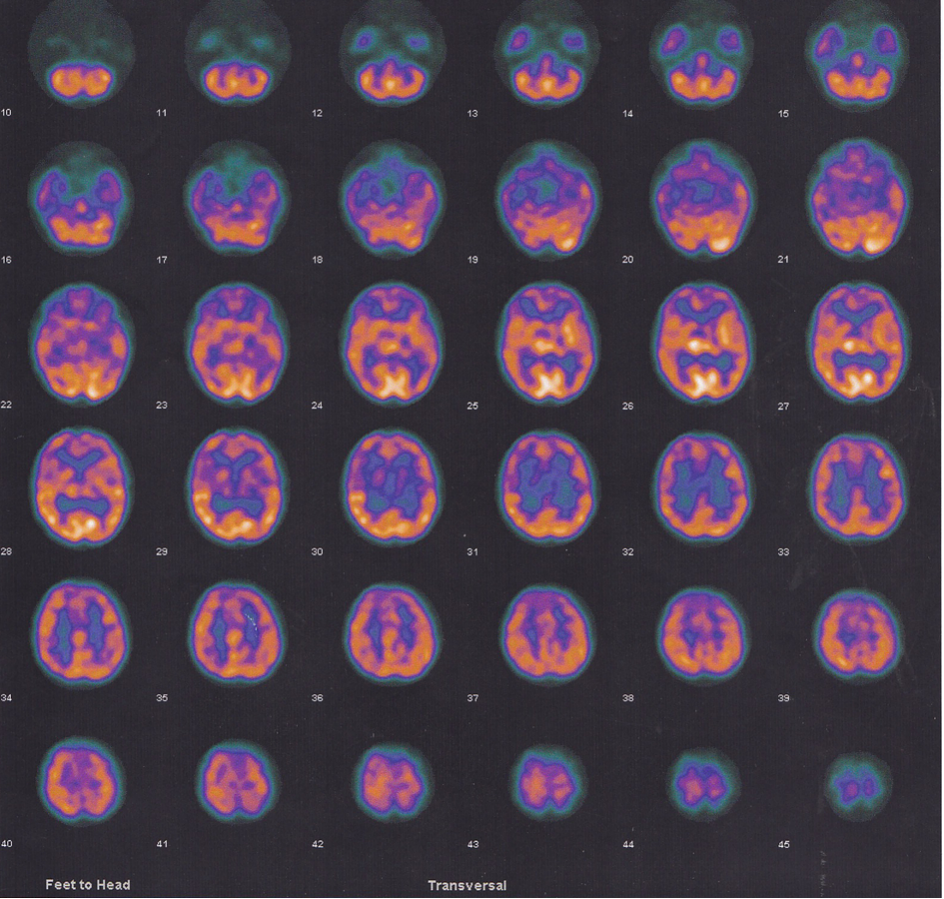
**Interictal brain SPECT with 99mTC-ECD showed a cerebral hypometabolism in both right and left parietal and occipital lobes**.

Interictal brain SPECT with 99mTC-ECD showed cerebral hypometabolism in both right and left parietal and occipital lobes (Figure 2).

**Figure 3:**
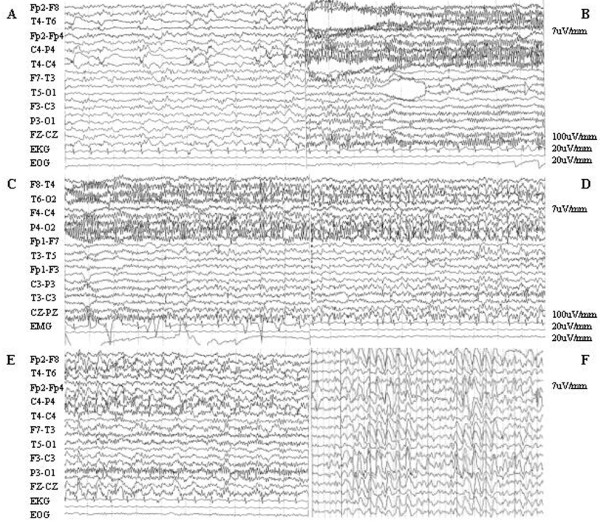
**Autoscopic seizures**. A: slow (3.5 Hz) right centro-parietal activity. Patient reported an unclear change in the awareness of her body, a feeling of strangeness. B: abrupt discharges constituted by polyspikes and sharp-waves of 100-120 uV in amplitude and in reversal phase at P4 lead. Patient reported a sudden appearance of her entire body exactly in front of her, in upright position. The double was at the same level of her real body, i.e. the real body was not felt elevated relatively to her double (unlike in out of body experience). She reported a bilocation state. The double was motionless and silent. C-D: after a few seconds the discharge involved right fronto-temporal channels and then left parieto-occipital channel. The discharge lasted about 30 seconds. We noted an impairment of consciousness. E: the final critic activity was constituted by right temporo-parietal spike and slow wave complexes (3.5-4 Hz). Patient reported again un unclear perception of her body, but the double had vanished. F: slow generalized interictal activity recorded by a previous EEG (3-3.5 Hz).

A previous interictal EEG showed sporadic posterior bilateral slow wave activity 3-3.5 Hz (Figure 3F). During her repeated evaluations the patient was instructed to signal verbally or by hand gestures the possible occurrence of her hallucinations. We recorded a video-EEG during one of her autoscopic experiences showing an epileptic activity (Figure 3) consisting of a brief (about 1 second) and slow (3.5 Hz) right centro-parietal activity followed by abrupt discharges represented by fast activity of polyspikes and sharp-waves of 100-120 uV amplitude and in reversal phase at the P4 lead. After a few seconds the discharge involved right fronto-temporal channels and then the left parieto-occipital channel. The discharge lasted about 30 seconds. The final critical activity was represented by a 3.5-4 Hz spike and slow wave complexes overlapping to widespread slow activity. No ictal automatisms or motor signs were present.

During the video-EEG recording, before the onset of the seizure, the patient reported an unclear change in the awareness of her body, with feelings of derealisation. Then she signalled by hand gesture the abrupt appearance of her entire body exactly in front of her, in upright position and in the same perspective of her previous experiences. This sensation was coincident with the seizure. During an a posteriori interview she reported the impression of bilocation. In this episode the double was motionless and silent. This view of herself in normal size, with the same clothing and facial expression was concomitant to the right centro-parietal discharge characterized by fast activity of polispikes and sharp-waves in reversal phase at the P4 lead. When the discharges involved right fronto-temporal channels we noted an impairment of consciousness: the patient, with fixed eyes, stopped answering our questions, even though she did not report clouding of consciousness in the a posteriori interview. No ictal automatisms or motor signs were observed. During the widespread slow EEG activity the patient reported again an unclear perception of her body, but she signalled that the autoscopic image had disappeared.

We aimed to study functional connectivity by means of functional magnetic resonance imaging (fMRI) [30], but the patient expressed her unavailability to further examinations.

We prescribed Levetiracetam (3000 mg/day) which resolved her he-autoscopic seizures. Levetiracetam was administered following the evidence of the seizures.

In the following two months the reported that the seizures did not recur, but depression was evidenced in repeated evaluations, with apathy, anaedonia and anxious agitation. We decided to address the patient to psychiatric care due to severe depressive symptoms and to the high risk of further suicidal attempts.

## Discussion and review of the literature

Autoscopic phenomena are illusory own body perceptions that affect the entire body and lead to conspicuous abnormalities in embodiment as well as in body ownership. Brugger [2] focused on the main features of the six types of autoscopic phenomena presenting a classification scheme based on phenomenological criteria. Depersonalisation symptoms are absent only in autoscopic hallucination and can be described as a reduced awareness of own body in negative heautoscopy, as a sense of empty body in inner heautoscopy, as a subjective meaningfulness and enhanced reality of the experience in out of body experience and as a sense of familiarity with the external presence in the feeling of a presence. Table 1 summarizes some features of the six types of autoscopic phenomena.

**Table 1:** Main features of different autoscopic phenomena.

	AH	h-A	FOP	OBE	Nh-A	Ih-A
**SEE ONE'S OWN BODY AS**	ONLY PARTS OF THE DOUBLE ARE SEEN IN EXACT MIRROR IMAGE	MIRROR IMAGE, MAINTAINED SIDEDNESS, DOUBLE IS COLOURLESS, ECHOPRAXIA BILOCATION	OFTEN ILLUSION OF THE PRESENCE AT THE FRINGE OF VISION, h-A WITHOUT OPTICAL IMAGE	SEPARATION FROM ONE'S OWN BODY AS THE CORE OF THE EXPERIENCE; FROM ELEVATED PERSPECTIVE	FAILURE TO PERCEIVE ONE'S OWN BODY EITHER IN A MIRROR OR WHEN LOOKED AT DIRECTLY	THE INNER ORGANS ARE VISUALLY HALLUCINATED IN THE EXTRAPERSONAL SPACE
**DURATION**	SECONDS/MINUTES	SECONDS/HOURS	SECONDS	SECONDS/MINUTES	SECONDS/MINUTES	SECONDS/MINUTES
**DISEMBODIMENT**	-	+/-	+/-	++	+/-	+/-
**MAINTAINED SIDEDNESS**	-	+	NA	+	NA	NA
**PSYCHOLOGICAL PHENOMENOLOGY**	-	++	+	++	-	-
**INVOLVED CEREBRAL CORTEX **	PO (RIGHT?)	TPJ (LEFT?)	?	TPJ (RIGHT?)	SPLENIUM (ONE DESCRIPTION)[69]	?
**PREDOMINANT INVOLVED MODALITIES**	VISUAL	VISUAL AND SOMAESTHETIC	SOMAESTHETIC ONLY	VISUAL AND SOMAESTHETIC	VISUAL AND SOMAESTHETIC	VISUAL AND SOMAESTHETIC
**ACCOMPANYING SYMPTOMS**	MICROPSIA HEMIANOPIA	D	D	D, SENSATION OF FLOATING OR FLYING, AUDITORY HALLUCINATIONS OR ILLUSIONS OF VIBRATION	D, ASCHEMATIA ASOMATOAGNOSIA	D
**VESTIBULAR DISTURBANCE**	-	+	-	++	-	-

AH = autoscopic hallucination, h-A = he-autoscopy, FOP = feeling of a presence, OBE = out-of-body experience, Nh-A = negative heautoscopy, Ih-A = inner heautoscopy, PO = parieto-occipital cortex, TPJ = temporo-parietal junction, D = depersonalitation, - = absent, + = present, NA = not applicable; ? = not known; maintained sidedness indicate no shift from left to right in the autoscopic image.

### Phenomenological aspects of he-autoscopy

We could report a case of he-autoscopy in which the affected patients have a vague feeling of detachment from the body accompanied by the sensation of a "double consciousness". He-autoscopy involves visual and somaesthetic modalities and, similar to out of body experience, localizes the self in an illusory body at an extra personal space although the point of localization of the ego remains within the natural boundaries of one's own body. There are some variables that can help to differentiate among autoscopic phenomena: the presence of vestibular hallucinations or body schema disturbances, the position of the autoscopic and the physical body, the kind of view, the sense of bilocation and finally the sharing of thoughts and words or actions of the autoscopic body. Among the described variables five phenomenological characteristics of the autoscopic body allow to distinguish he-autoscopy from the other abnormal experiences:

1) the "view": in he-autoscopy subjects see the autoscopic body in front-, side- or back-views.

2) the "actions": the autoscopic body can act only in he-autoscopy (activities of the autoscopic body appear to be specific to he-autoscopy and almost absent in out of body experience).

3) the experience of "sharing of thoughts and words" which are often associated with he-autoscopy and are less frequent in out of body experience.

4) the "perspective": in he-autoscopy patients frequently experienced to see the double from several different visuo-spatial perspectives that, in contrast, were unequivocally localized and experienced as unitary by all out of body experience patients.

5) the "bilocation": only he-autoscopy subjects reported to be split into two parts of selves.

In addition positive and neutral emotional experiences are especially rare in he-autoscopy [12,17].

Thus he-autoscopy significantly differs in these complex variables from other autoscopic phenomena, suggesting that different central mechanisms are underlying. The phenomenological variability of the autoscopic body (with respect to views and actions) and the increased frequency of shared thoughts, voices, and actions between autoscopic and physical body might be due to different involvements of kinesthetic/proprioceptive information processing in he-autoscopy. The sharing of thoughts, voices and actions might make difficult for the patient to decide where the physical agent is located and leads to the experience of two observing selves. Therefore he-autoscopy is not only the reduplication of one's physical body, but also the reduplication of one's self. The terrifying experience of "not knowing where the self is" could justify the association with suicide attempts, as in our patient. Our case can be considered as an example of he-autoscopy in which, typically, the autoscopic body acts exactly like the real body (maintaining sidedness) and interacts with it: the patient experienced to have access to the autoscopic body's thoughts and sometimes could verbally communicate with it. Typically the patient had the impression of being at two locations at the same time (bilocation) and positive or neutral emotions never accompanied her autoscopic experience. She also experienced the he-autoscopy in the typical upright position, while the view of the self always in front of her (as in out of body experience) in the same visuo-spatial perspective, is less commonly reported [2].

Vestibular sensations were not reported by the patient: in the Blanke and Mohr analysis [31], vestibular hallucinations and body schema disturbances are comparatively frequent in out of body experience and he-autoscopy (about 60% of patients). Our case report suggests that vestibular symptoms are not specific of he-autoscopy and that their absence does not exclude he-autoscopy or out of body experience.

### He-autoscopy, epilepsy, depression and suicide

Psychological phenomenology differs among different autoscopic experiences: he-autoscopy and out of body experience are experienced as profoundly significant events and in he-autoscopy the quality of emotional impact considerably varies: one's doppelgänger can sometimes be experienced as highly supportive [14], but in general is offensive or overtly aggressive. A classical case was reported by Wigan [32], who described the first non-fictional account of a man who could induce a visual hallucination of himself. Gradually the double became more and more autonomous appearing without being evocated. Utterly distraught, the man shot himself. Significantly our patient attempted suicide twice, but specific risks linking he-autoscopy and suicide are not statistically supported. The association between suicide and he-autoscopy is a common theme in dramatic romances and the appearance of the doppelgänger, a ghostly double of a living person, often announces the hero's death that is usually a death by suicide. In Breton myths the doppelgänger is a version of the Ankou, a personification of death; in a tradition of the Hebrew Talmud, to meet himself means to meet God. There are also several literary examples: in Dostoyevsky's novel "The Double" when the protagonist experienced his first autoscopic experience, he began to think to kill himself by drowning. In Edgar Allan Poe's short story "William Wilson", probably the most dramatic image, the main character, of questionable morality, is dogged by his doppelgänger most tenaciously when his moral fails. But also Oscar Wilde ("The portrait of Dorian Gray"), Franz Werfel ("Spiegelmensch") and Friedrich von Gerstäcker ("Der doppelgänger") had their heroes commit suicide to escape the horror of being haunted by their second selves. Some of these authors have not only had epilepsy but have also known he-autoscopy from personal experience [33,34]. There are several notable reports about documented autoscopic experiences: Percy Bysshe Shelley, an English poet, drowned in the Bay of La Spezia near Lerici and Abraham Lincoln, known to be a superstitious man, recognised a special meaning to an autoscopic experience which he seems to have had in the evening of his election [35]. A rare and dubious example of a doppelgänger which was both benign and reassuring is described by Johann Wolfgang von Goethe in his autobiography "Truth and Fiction" ("Dichtung und Wahrheit"). More recently Otto Rank, a Freudian Austrian psychoanalyst, in his book "The Double" [36], suggested a possible explanation for the frequent recurrence of the image of the double in the artistic production and folklore. He believes that the double is an artist's strategy to exorcise the terrible idea of death. "Heautoscopic suicide" also includes cases of attempts to kill one's double or, in the most obviously transitivistic manner, observing one's doppelgänger committing suicide [37].

Keppler observed that "Often the conscious mind tries to deny its unconscious through the mechanism of "projection", attributing its own unconscious content (a murderous impulse, for example) to a real person in the world outside; at times it even creates an external hallucination in the image of this content" [14].

Brugger examined the "hostile interactions between body and self" [38] indicating four major variants of "heautoscopic suicide" as an actively imposed or passively experienced form of self-injurious behaviour: self-injury/suicide in an attempt to escape the double, fenestration in order to get rid of the double, self-injury/suicide claimed to be inflicted by the double, self-injury/suicide in an attempt to kill the double and the observation of the doppelgänger's self-injury/suicide. He discussed neuropsychiatric and psychodynamic approaches to disembodiment concluding that, although there is no direct clinical or neuroanatomic evidence for a primary callosal pathology, it is not entirely implausible to assume an inter-hemispheric disconnection at the basis of heautoscopic aggression. Of note, in our patient an early MRI study reported the presence of enlarged splenium callosi. Our MRI study however, including tractography examination, did not evidence any abnormality. Our measurements were, in our patient, inside the range reported for female subjects, who have commonly larger splenial bodies than males [39,40]. Even though heautoscopy, epilepsy and suicide may be a more common triad than previously recognized [41], we know little about the mutual correlation, but the description of the survivors, as in our case, would rather favour a causal relation between the double experience and the impulse to kill oneself. Suicide attempts are not infrequent in depressed subjects and depression is commonly associated with epilepsy [42]. In severely depressed patients "heautoscopy of the Cotard type" (seeing a dead doppelgänger during the hallucinatory attendance of one's own funeral) is not a rarity. The suicidal risk is further increased by psychiatry comorbidity and drug use [43-45]. The epileptic focus in the right hemisphere and the severe depressive symptoms presented by our patient, support recent hypothesized links between depression and inter-hemispheric imbalance. Depression is believed to be associated with a hyperactive right-hemisphere and a relatively hypoactive left-hemisphere [46]. Yet, the underlying mechanisms which can explain the involvement of the right side remain elusive. There is evidence that the right hemisphere is selectively involved in processing negative emotions, pessimistic thoughts and unconstructive thinking styles. Additionally, it mediates vigilance and arousal and had also been linked with self-reflection, accounting for the tendency of depressed individuals to withdraw from their external environments and focus attention inward [46]. The involved hemisphere is also important to explain possible language deficits, but ictal speech disorders were not present in our patient in agreement with the involvement of non dominant hemisphere as showed by EEG recording. Word finding difficulties are often reported by epileptic patients and peri-operative and post-surgical electro-cortical stimulation evidences have highlighted a role for the anterior part of the dominant temporal lobe in oral word production [47]. In previous reviews [23,48,49], autoscopic experiences are mentioned as a rare but classic symptom in parietal lobe epilepsy. However it has been reported in association with temporal lobe epilepsy [50]. There are few reported cases of postictal autoscopic phenomena [51]. Autoscopic experiences have been described not only in focal seizures, but in a broad range of neurological disorders such as migraine, neoplasia, infarction and infection [11,12,52] and also in psychiatric disorders such as schizophrenia, depression, anxiety and dissociative disorders [16,17]. In some cases it is difficult to distinguish the autoscopic experiences from a dreamy-state in which the self is seen in more complex, dream-like or memory-like scenes without actually seeing the image of one's face or body [53].

Our patient experienced typical he-autoscopy and the autoscopic episodes were the only ictal symptom. The ictal EEG performed during the autoscopic experience, showed a right parietal origin in non-dominant hemisphere, with rapid bilateral posterior involvement. These data were confirmed by SPECT findings that revealed a hypometabolism in parietal and occipital areas but no brain abnormality was detected by means of MRI. In contrast with neurophysiological data, patient reported neither macroasomatognosia, a symptom that can result from an involvement of the parietal lobe [23,48,49], nor palinopsia (consistent with an involvement of the visual associative area) nor luminous flashes in the visual fields (consistent with an involvement of the primary visual area) nor motor symptoms.

### Possible cerebral mechanisms: multisensory processing and cortical hyperconnectivity

Defining the mechanism of autoscopic phenomena is a challenge. Menninger-Lerchental [13] first speculated on different anatomical substrates suggesting that autoscopic hallucination originate at the junction of the parietal and occipital lobe, he-autoscopy from the angular and supramarginal gyrus and out of body experience from the superior parietal lobe. Blanke and Mohr [31] summarized anatomical findings of their analysis evidencing that he-autoscopy seems to primarily involve the left temporo-parietal junction and out of body experience the right. More recently Blanke and Arzy [54] have unexpectedly reproduced an effect strongly reminiscent of the doppelgänger phenomenon via electromagnetic stimulation of the left temporo-parietal junction. Their analyses showed that out of body experiences and he-autoscopy are primarily associated with electrical stimulation, probably inhibition, of the temporo-parietal junction and autoscopic hallucination with electrical stimulation in parieto-occipital cortex. Moreover these experimental data suggest that out of body experiences are associated with damage to the right temporo-parietal junction and he-autoscopy to the left temporo-parietal junction[31,54], whereas autoscopic hallucination is associated with damage to the right parieto-occipital cortex [55]. Corporal awareness and experience of the body ownership are more likely dependent on the right hemisphere [28]. However with regard to hemispheric asymmetries no firm conclusion can be drawn. Our case shows an epileptic focus in the right temporo-parietal junction: the patient report suggests that the hallucination perception was an he-autoscopy, as it presents most of the peculiar characteristics reported by literature in he-autoscopy. Therefore our report might suggest that he-autoscopy can possibly arise also from epileptic foci of the right hemisphere. The epileptic discharge (Figure 3A-E) shows a definite localization and the involvement of the left hemisphere occurs only when the patient subjectively reports that the hallucinatory image has disappeared. In our case report, therefore, the EEG shows an epileptic discharge that originates in the right temporo-parietal junction. It might be argued that the only evidence of the he-autoscopy corresponding to EEG discharge is obtained from the patient, subjectively signalling the presence of autoscopic image of the self during the seizure and by her detailed description "a posteriori". Thus exact timing of the different feelings might appear uncertain: nonetheless EEG discharge was observed and he-autoscopy was reported. We believe it would be difficult to obtain better evidence then the one we are reporting, as a verbal description of symptoms during the seizure would alter the EEG due to glosso-kinetic artefacts.

Several studies [11,12,31,54-56] proposed that autoscopic phenomena are a double failure to integrate proprioceptive, tactile and visual information with respect to one's own body (disintegration in personal space), along with an additional vestibular dysfunction leading to disintegration between personal (vestibular) space and extrapersonal (visual) space. Disintegration of personal space is present in he-autoscopy and out of body experience but differences are mainly due to the level and type of vestibular dysfunction. In more detail: out of body experiences are associated with specific vestibular, graviceptive and otholithic sensations that are characterized by a variety of sensations including feeling of elevation and floating, he-autoscopy is associated with a moderate and more variable vestibular disturbance while no vestibular disturbance is evidenced in autoscopic hallucination, feeling of a presence, negative heautoscopy and inner heautoscopy. Moreover, the high frequency of visual hallucinations and hemianopia in patients with autoscopic hallucination suggests that defective visual processing of bodily information is the main causing factor for disintegration in personal space in autoscopic hallucination.

Therefore according to Blanke we speculated that the different forms of autoscopic phenomena are related to different degrees of vestibular dysfunction. We hypothesize that autoscopic seizures occur because of ictal disturbances of the normal integration process of body representation within parieto-occipital networks in which the right inferior parietal region plays a significant role. The integration of proprioceptive, tactile and visual information with respect to one's body with vestibular information is important for the constant updating of the movement and position of single body parts and entire body. In order to create a central representation of one's own body, the brain must integrate and weigh the evidence from different sensory sources.

Electrophysiological [57] and neuroimaging [27,29] studies showed that the vestibular cortex is a multisensory cortex receiving not only vestibular information, but also visual, proprioceptive and tactile cues from the whole body. We believe that these multisensory interactions are fundamental for integrating signals about body movements and body position in space with respect to other body segments and to the ground. The implication of temporo-parietal junction in embodiment and in self processing is suggested by neuroimaging studies in healthy subjects [29,58-60]. These studies examining visuo-spatial pathways showed the involvement of many other cortical areas such as prefrontal, parietal and temporal cortex. The "cortical midline structures" have been recently identified as intimately related to the experience of the self [61]. Therefore the temporo-parietal junction might be a crucial structure for conscious experience in a widely distributed network of cortical areas.

In conclusion it is not known which of the many senses are primarily involved in the generation of he-autoscopy. Data in literature shows that visual theories cannot entirely explain autoscopic phenomena and points to the importance of non visual, body-related, mechanisms such as proprioceptive and/or kinaesthetic processing. A further aspect in favor of tactile and proprioceptive mechanisms was given by the different body positions reported by patients, upright for he-autoscopy and supine for out of body experience, suggesting differential influences of proprioceptive and tactile processing in different types of autoscopic experiences. Blanke and Mohr [31] summarized anatomical findings of their analysis evidencing that he-autoscopy seem to primarily involve the left temporo-parietal junction and out of body experience the right. The evidence of the temporal lobe involvement in body-distortion processing and impairments in own-body transformations was highlighted in a recent report [62]. Yet self-representation has been shown to depend also on the prefrontal cortex and its connectivity with temporo-parietal junction[63-66]. Accordingly, we believe that he-autoscopy might not only be mediated by the temporo-parietal junction but also depend on frontal lobe functioning and processing and the diffusion of the discharge on the anterior channels during the recorded autoscopic seizures corroborates this idea (Figure 3C-D). A putative implication for fronto-parietal connectivity also in out of body experience was recently proposed [67]. Others authors [68] stress the visual modality of these phenomena and hypothesize a process of cognitive dedifferentiation in which visual hallucinations are derived from available non-visual sensory cues through an underlying hyperconnectivity of cortical structures regulating vestibular and visual representations of the body and those responsible for the rotation of environmental objects. Probably a specific cortical network is involved in the perceptions of body into space and studies of cerebral connectivity during vestibular stress could be an helpful and interesting approach to a better understanding of autoscopic phenomena. Because of the severe psychiatric symptoms presented in our patient, we could not perform the combined EEG/fMRI analysis that recently allowed to identify the cortical connection network sustaining persistent symptoms in epilepsy [30], yet we believe that this approach might be ideally suited to understand the mechanism of autoscopic phenomena.

## Competing interests

**Financial competing interests: **The authors declare that they have no competing financial interests.

**Non-financial competing interests: **The authors declare that they have no competing non-financial interests.

## Authors' contributions

FA performed clinical evaluations and prepared the manuscript. MO provided the case and designed the overall paper. VO, LB and AT revised the manuscript for important intellectual content and provided bibliographical research. VM, LR, RF were involved in analysing EEG, MRI and SPECT data and in providing technological support. All authors read and approved the final manuscript.

## Consent

Written informed consent was obtained from the patient for publication of this case report and accompanying images. A copy of the written consent is available for review by the Editor-in-Chief of this journal.
